# QR code security: an adaptive retraining approach for dynamic URL-based threat detection

**DOI:** 10.1038/s41598-026-43002-z

**Published:** 2026-04-22

**Authors:** Hissah Almousa, Suliman A. Alsuhibany

**Affiliations:** Department of Computer Science, College of Computer, Qassim University, Buraydah, 51452 Saudi Arabia

**Keywords:** Cybersecurity, QR code, Adaptive retraining, BERT-based classification, Malicious URL detection, Attention mechanism, Computational biology and bioinformatics, Engineering, Mathematics and computing

## Abstract

The extensive integration of QR codes into daily activities highlights their pivotal role in facilitating seamless information exchange. However, this widespread adoption also exposes QR codes to cybersecurity threats. Malicious QR codes, often persuasive as legitimate code, can carry unwanted or harmful content, posing significant risks to users. Particularly, the encoded information within QR codes, including URLs, is a prime target for cybercriminals. Consequently, to effectively mitigate these threats, precise detection systems are imperative for identifying novel instances of malicious URLs embedded within QR codes. Traditional approaches, like blacklists, are increasingly inadequate against the evolving threat landscape. Therefore, there has been a shift towards leveraging pre-trained language modelling techniques as a more robust solution to enhance QR code security. This study aims to harness the power of a BERT-based architecture to differentiate and classify QR codes based on the URLs embedded within them, distinguishing between benign and malicious URLs. Moreover, the study adopts an adaptive and dynamic approach through periodic model refinement. By continuously updating and retraining the model with newly recorded URL data sourced from diverse resources, this methodology ensures its adaptability to evolving threats, thus fortifying QR code security measures. The experimental findings from the proposed BERT model showcase superior performance in accurately detecting malicious URLs, outperforming other methods.

## Introduction

Quick Response (QR) codes, introduced by Denso Wave in 1994 for vehicle identification, have become widely used across various industries due to their versatility and convenience^1^. Their popularity surged, particularly during the COVID-19 pandemic, leading to a 57% increase in scans between 2021 and 2022, according to Uniqode, with over 32 million scans recorded^2^. This widespread adoption emphasizes their significant role in connecting the physical and digital realms.

Despite their widespread utility, QR codes have also become a target for cybercriminals, who exploit their convenience to embed malicious URLs, leading to significant security risks such as malware distribution and phishing attacks^3^. The escalating frequency of such URL-based attacks necessitates a closer examination of the underlying motivations, particularly the human and psychological factors that make these attacks successful^4^. Studies, such as those by Krombholz et al.^5^, reveal that the inherent design of QR codes, facilitating easy access to information, also makes them vulnerable to various cyber threats.

The distinction between the physical QR code and its embedded content is essential. This study delimits its scope to URL-embedded threats, excluding physical or visual tampering. The QR symbol merely serves as a passive data carrier (ISO/IEC 18004:2024), while the URL constitutes the actionable component representing a scalable, remote attack vector.

Traditional methods for detecting malicious URLs, which often involve machine learning and manual feature extraction, have shown some effectiveness but are limited by their reliance on pre-processed data and historical patterns^6,7^. These methods struggle to adapt to new and emerging threats in real time, a limitation that has spurred interest in more dynamic approaches. Deep learning models, particularly those employing character embeddings, offer an enhanced capability to detect malicious URLs by analyzing the embedded content of QR codes. However, these models face challenges such as interpreting character positions within URLs and managing the relationships between characters, which can impact their overall effectiveness^8^.

The integration of advanced deep learning techniques, including Recurrent Neural Networks (RNNs) and Convolutional Neural Networks (CNNs), has shown promise in improving the accuracy of malicious QR code detection by identifying patterns and anomalies that traditional methods might miss^9,10^. Building on this, recent research has explored transformer-based models, such as BERT, for detecting malicious URLs, offering a more nuanced approach to security^11,12^. However, these models are often constrained by their initial design, which may not adequately address the dynamic nature of evolving cyber threats.

In response to these challenges, this paper proposes an adaptive BERT-based method for detecting malicious URLs in QR codes, involving iterative model updates with new data to address emerging threats. By combining advanced deep learning with adaptive retraining, this approach enhances cybersecurity and ensures the model remains effective against evolving threats.

The key contributions of this research are:

1. Ensure a secure user experience by effectively assessing QR codes for safety, distinguishing between legitimate and malicious codes.
2. Fine-tune the BERT for enhanced real-world adaptability and accurate detection of diverse URL patterns.
3. Assess the robustness of the developed BERT model against a diverse set of benign and malicious URLs.
4. Conduct a comparative analysis with baseline methods to establish the superiority of the proposed adaptive URL-based approach for QR code security.

The remainder of this paper is organized as follows: The Literature Review section summarizes prior work relevant to this study. The Methodology section outlines the model framework and proposed URL-based strategies. The Model Implementation section outlines the training, adaptive retraining, and testing phases, as well as the setup of experiments and the evaluation of outcomes, which are explained in the following subsections. The findings are presented in the Experimental Results Analysis section, followed by the Discussion section. Finally, the Conclusion section offers concluding remarks for the study.

## Literature review

The classification of malicious QR codes involves methods that rely on cryptographic techniques for verification and security. These techniques, which include encryption, digital signing, and access management, are crucial for ensuring confidentiality and privacy^13^.

Kelvin and Kang Leng^14^ emphasize digital signing, encryption, and user education, with third-party detectors suggested for enhanced protection. Bani-Hani et al.^15^ developed a protected QR code framework using Digital Signature Algorithm (DSA) and hashing for data integrity. Mavroeidis and Nicho^16^ introduced QRCS, a cryptographic tool for QR code verification using hash functions and digital signatures, blocking codes if verification fails.

Hegde et al.^17^ proposed AMPS, a scanner with built-in malicious content detection and AES encryption. Wahsheh and Luccio^1^ created BarSec, employing symmetric and asymmetric cryptography for QR code security. However, challenges like insufficient key lengths, vulnerable encryption algorithms, and the need for the same application for generating and reading cryptographic QR codes hinder widespread adoption^18^.

The need to classify unsafe QR codes and their embedded malicious URLs, often used in phishing and malware attacks, is critical. Various studies address this issue using Machine Learning (ML) and Deep Learning (DL) techniques.

Do Rosario and Lourenco^3^ used machine learning models like Random Forest (RF) and Support Vector Machine (SVM) to classify URLs as either malicious or benign, achieving up to 96% accuracy. They noted issues such as limited datasets and feature engineering challenges. Similarly, Gogoi et al.^19^ proposed a hybrid method combining blocklists and machine learning, reaching accuracies of 97% and 98% for shallow and deep models, respectively. Kumar et al.^20^ introduced a multi-layer filtering technique with Naive Bayes (NB), Decision Trees (DT), and SVM, achieving 79.5% accuracy.

In contrast, Prabakaran et al.^21^ introduced an advanced phishing detection method using DL, combining Variational Autoencoders (VAE) with Deep Neural Networks (DNN) for feature extraction and classification, achieving 97.8% accuracy. Thakur et al.^22^ explored DL techniques to improve the classification of malicious URLs, employing methods like TF-IDF vectorization and N-grams for feature extraction, and achieving an accuracy of 96.9%. Recent research has introduced a hybrid framework combining visual watermarking with CNNs to verify the authenticity of printed QR codes with 97.2% accuracy^23^.

Further advancing the field, Sen et al.^24^ emphasized the role of DL and Natural Language Processing (NLP) in cybersecurity, achieving 99.6% accuracy with a hybrid CNN-LSTM model. They demonstrated that character embeddings, rather than word embeddings, yield higher accuracy in detecting malicious URLs. Chen et al.^25^ introduced a multilayer recurrent convolutional neural network model, augmented with the YOLO algorithm, to detect malicious URLs, achieving a 94.8% accuracy rate. Rakotoasimbahoaka et al.^26^ explored a majority vote approach, integrating various DL and ML models such as RF, LSTM, and CNN, to achieve a 93% accuracy rate.

Mumu et al.^27^ explored the use of both DL and ML algorithms on a Kaggle dataset, employing classifiers like RF and a Feed-Forward Neural Network (FNN). The RF classifier achieved 91.4% accuracy, while the FNN, optimized with the Adam algorithm, reached 95.9% accuracy. Pinerio and Portillo^28^ introduced a web architecture that uses ML for detecting phishing URLs, which became more prevalent during the COVID-19 pandemic. Their system, based on RF and SVM models, achieved 80% accuracy. Alsaedi et al.^29^ addressed the challenges associated with web-based content in identifying phishing threats by proposing a Cyber Threat Intelligence-based Malicious URL Detection Model (CTI-MURLD). This approach, employing ensemble learning and integrating features from cyber threat intelligence, attained an accuracy rate of 96.8% and significantly minimized false positives. Almousa et al.^30^ introduced QR Shield, a hybrid ML model that enhances QR code security by detecting malicious URLs, achieving a 96.8% accuracy rate.

The application of NLP and transformer-based models to malicious URL detection has also been explored extensively. Xu et al.^11^ developed a DL architecture utilizing transformers to identify phishing URLs, which outperformed six classical models with a 97.3% detection accuracy. Ming and Kuan^12^ further advanced this field by introducing a BERT-based model for malicious URL detection, achieving accuracy rates of up to 98.7% across multiple datasets. Rahali and Akhloufi^31^ proposed MalBERT, a malware classification method that utilizes Transformer technology, achieving high accuracy rates in binary and multi-class malware classifications.

In phishing detection, Singh et al.^32^ implemented a DL-based phishing threat detection system through CNNs, reaching 98% accuracy without requiring manual feature engineering. Ariyadasa et al.^33^ introduced PhishDet, employing Graph Convolutional Networks (GCN) and Long-term Recurrent Convolutional Networks (LRCN) to achieve a 96.4% accuracy rate in detecting phishing websites, though they emphasized the need for periodic retraining to maintain performance. Other recent study has addressed infrastructure-level QR threats by leveraging anomaly detection, risk signaling, and four ML models to identify diverse attack vectors such as payload injection and exfiltration with over 96% accuracy^34^.

According to the reviewed literature, DL and transformer-based models have emerged as promising alternatives to traditional methods for addressing security challenges. The proposed model aims to integrate these advancements, building on key findings to enhance the fight against digital exploitation and cybercrime. To provide a clear overview of all studies, Table 1 categorizes research according to their respective approaches, dataset employed, and accuracy results.

**Table 1:** Explored malicious QR codes solutions.

		Approach					Dataset					Accuracy
Solutions	Year	Cryptography	Blacklisting	Machine Learning	Neural Networks	Transforms models	Kaggle	ISCX-URL-2016	Phish-tank	GitHub repositories	Other resources	
^15^	2014	$\checkmark$									$\checkmark$	NA
^20^	2017			$\checkmark$							$\checkmark$	79.5%
^16^		$\checkmark$									$\checkmark$	NA
^17^	2018	$\checkmark$									$\checkmark$	NA
^14^	2019	$\checkmark$									$\checkmark$	NA
^26^	2020			$\checkmark$	$\checkmark$						$\checkmark$	93%
^32^					$\checkmark$						$\checkmark$	98%
^1^		$\checkmark$									$\checkmark$	NA
^24^	2021				$\checkmark$				$\checkmark$		$\checkmark$	99.6%
^25^					$\checkmark$						$\checkmark$	94.8%
^11^						$\checkmark$			$\checkmark$		$\checkmark$	97.3%
^35^						$\checkmark$					$\checkmark$	99.6%
^31^						$\checkmark$					$\checkmark$	97.6%
^19^	2022		$\checkmark$	$\checkmark$					$\checkmark$	$\checkmark$		98%
^22^					$\checkmark$		$\checkmark$					96.9%
^28^				$\checkmark$					$\checkmark$		$\checkmark$	80%
^29^				$\checkmark$	$\checkmark$				$\checkmark$		$\checkmark$	96.8%
^36^				$\checkmark$					$\checkmark$	$\checkmark$		98.9%
^33^					$\checkmark$						$\checkmark$	96.4%
^3^	2023			$\checkmark$							$\checkmark$	96%
^21^					$\checkmark$		$\checkmark$	$\checkmark$	$\checkmark$			97.8%
^27^				$\checkmark$	$\checkmark$		$\checkmark$					95.9%
^12^						$\checkmark$	$\checkmark$	$\checkmark$		$\checkmark$		98.7%
^30^	2024			$\checkmark$			$\checkmark$					96.8%
^37^					$\checkmark$			$\checkmark$	$\checkmark$		$\checkmark$	99%
^23^	2025				$\checkmark$						$\checkmark$	97.2%
^34^				$\checkmark$			$\checkmark$		$\checkmark$			96%

## Methodology

To address the limitations identified in the literature, a dynamic methodology using BERT is proposed for detecting malicious URLs in QR codes. The proposed approach involves iterative retraining with new data from various sources to adapt to emerging threats. This section details the methodology for analyzing URLs in QR codes and developing a model to identify benign and malicious codes.

### Model framework

**Fig. 1:**
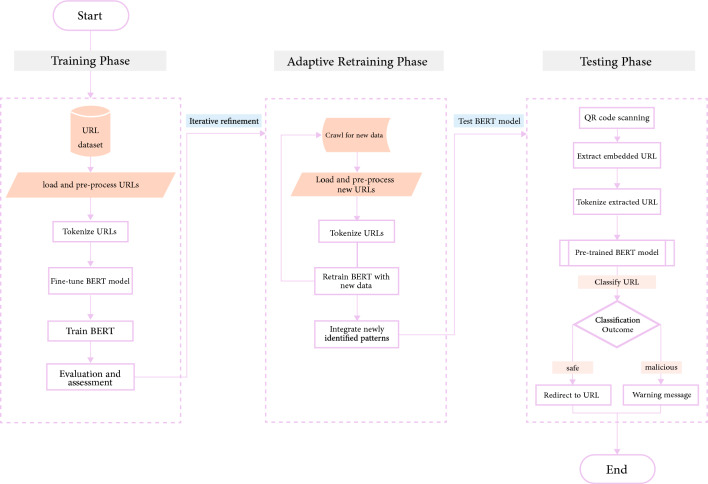
Abstract model framework.

The model framework section outlines the process of training and utilizing the BERT model to distinguish between benign and malicious URLs embedded within QR codes. The model follows a structured approach from training to testing, including the phase of adaptive model updates, as illustrated in Fig. 1.

The model undergoes an initial training phase using a diverse dataset of benign and malicious URLs from Kaggle, which are tokenized and converted into numerical representations for BERT. After fine-tuning and training, the model’s performance is evaluated for accurate classification. To address evolving threats, the model incorporates periodic model refinement by adding recent malicious URLs from sources like URLhaus and retraining to adapt to new attack patterns. In the testing phase, QR codes are scanned to extract URLs, which are then tokenized and classified by the pre-trained BERT model. Users are redirected to safe web pages or receive warnings if malicious URLs are detected, thus reducing cybersecurity risks.

### Decoding QR codes

The process of URL retrieval initiates the implementation of testing the model, focusing on extracting the encoded URL within the QR code. Utilizing OpenCV and Zbar for QR code image processing, typically implemented in Python libraries such as cv2 and pyzbar. This involves capturing the QR code image, pre-processing it for clarity, and applying the decoding algorithm to extract the URL string. The extracted URL serves as the basis for further analysis and model testing, forming a critical step in the research process.

### Dataset

In this paper, the dataset undergoes two phases of model implementation: one for initial training and another for the retraining phase, employing adaptive retraining techniques. A dataset, sourced from Kaggle^38^, is used to train the BERT model. A subset of the dataset, containing over 20,000 unique URLs, comprising 10,075 benign URLs and 9,925 malicious URLs, was selected for further examination. This dataset serves as the foundation for training the model and evaluating its performance.

The dataset comprises two essential columns, as demonstrated in Table 2, which shows an example set from the datasets: The first column, labeled ’url’, contains a list of URLs representing various web addresses. The second column, labeled ’type’, denotes the class of each URL, distinguishing between the ’benign’ and ’malicious’ categories.

**Table 2:** Sample dataset entries sourced from Kaggle.

Url	Type
https://www.prebleny.com/?p=13556	Benign
jsoundstudio.tripod.com/index.html	Malicious
http://www.recyclemyit.co.uk/modules/www/santander.com.br/seguro/?jsp=c	Malicious
https://www.hunch.com/item/hn_3165864/captain-wild-bill-kelso/	Benign
www.kilowattsoftware.com/rooPage.htm	Malicious
mp3raid.com/music/krizz_kaliko.html	Benign
lookatmyownmatchpictures.com	Malicious
amazon.com/Marilyn-Manson-Dead-World-VHS/dp/1573623806	Benign

The second phase of training involves adaptive updates, where newly recorded data from sources such as URLhaus^39^ is incorporated. URLhaus provides updated malicious URLs every 5 minutes in diverse formats, including .csv and .json. The malicious URLs on URLhaus are verified as valid by the community and authenticated users. The malicious dataset provided by URLhaus encompasses a heterogeneous mix of active threats, including malware distribution sites, phishing links, and Command and Control (C2) servers. This diversity ensures the model is evaluated against a realistic, high-entropy threat landscape rather than a single attack vector. Since URLhaus only provides malicious URLs, the data is processed and labeled accordingly. The model is regularly updated with this real-time data, improving its ability to accurately classify URLs and protect users against malicious content.

### Data pre-processing

The data pre-processing stage starts with loading the labeled URLs from a CSV file and analyzing the dataset’s structure, including entry count, columns, and data types. The dataset is shuffled to ensure randomness and prevent bias. During retraining phase, new URLs are categorized, integrated with the original dataset, and cleaned of duplicates. The SMOTE algorithm addresses any dataset imbalances. Finally, the DistilBERT tokenizer converts each URL into numerical tokens, preparing the data for the BERT model.

## Model implementation

This section covers the practical steps in developing and evaluating the model, including training on a diverse dataset with URL strings and BERT tokenization, fine-tuning, and adaptation through periodic model refinement. It concludes with the testing phase, which demonstrates how the model can effectively classify QR codes and identify malicious content, illustrating its potential real-world application.

**Fig. 2:**
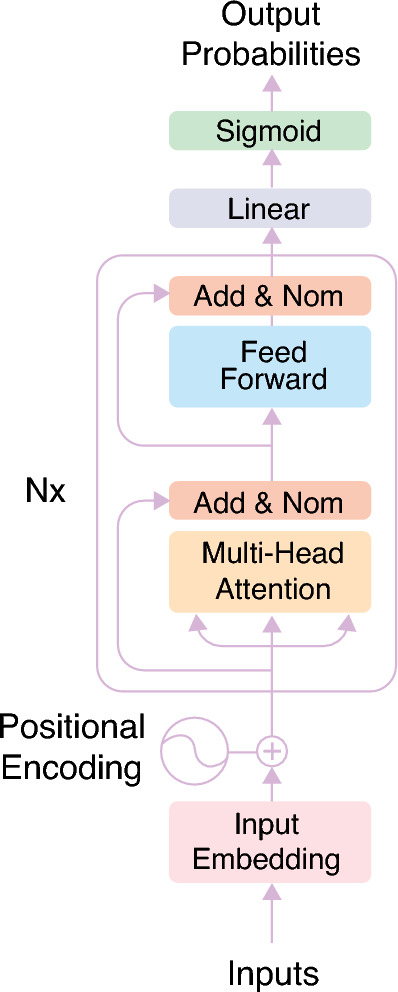
BERT architecture.

Figure 2 illustrates the inclusive architecture of BERT. Initially, input tokens are tokenized and converted to embeddings, processed through encoder layers with self-attention and FNNs to capture bidirectional context. The encoded representations are then linearly transformed and passed through a sigmoid function to produce the model’s final predictions.

### Training phase

Pre-trained BERT models, initially trained on large text corpora for tasks like Masked Language Model (MLM) and Next Sentence Prediction (NSP)^40^, greatly enhance classification accuracy when fine-tuned. Accessible via libraries like Hugging Face’s Transformers, BERT offers a powerful resource for improving classification tasks without further pre-training.

#### BERT tokenization: URL strings pre-processing

The dataset contains URL strings with labels, where URLs are tokenized using BERT. Unlike conventional character-level tokenization, BERT tokenization captures the contextual and semantic meaning of URLs by segmenting them into tokens. The DistilBERT tokenizer encodes and truncates URLs to 512 tokens, padding them for uniformity, enabling BERT to accurately classify benign and malicious URLs. Furthermore, features are extracted from tokenized URLs using a pre-trained DistilBERT model, which generates embeddings for each token. These embeddings serve as features for training the subsequent classification model.

**Fig. 3:**
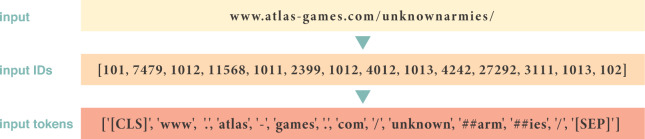
Tokenizing an example URL string utilizing BERT.

#### Fine-tuning: BERT model refinement

To better handle URLs, which are unnatural language, the pre-trained BERT model was enhanced utilizing transfer learning with the bert-base-uncased model. Uncased models are particularly advantageous for scenarios where word casing doesn’t hold significant semantic relevance, such as in URLs.

**Fig. 4:**
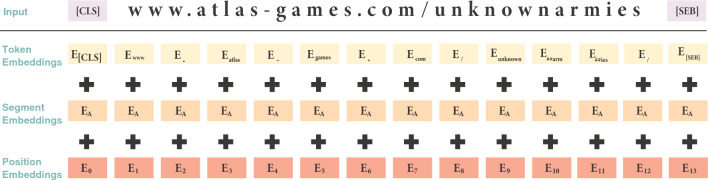
BERT input representation.

Three embeddings form the input for the BERT model, including token, segment, and position, as illustrated in Fig. 4. Token embeddings represent URL segments (e.g., the URL in Fig. 3 is split into 14 tokens). The [CLS] token indicates the beginning, and [SEP] tokens denote segment or sequence endings.

Segment embeddings differentiate between sentences A and B, but this distinction is irrelevant here as the URL is treated as a single sentence. Position embeddings indicate each token’s position. Since the URL is a single sentence, all tokens receive attention, resulting in all elements of the attention mask tensor to be assigned a value of 1.

**Fig. 5:**
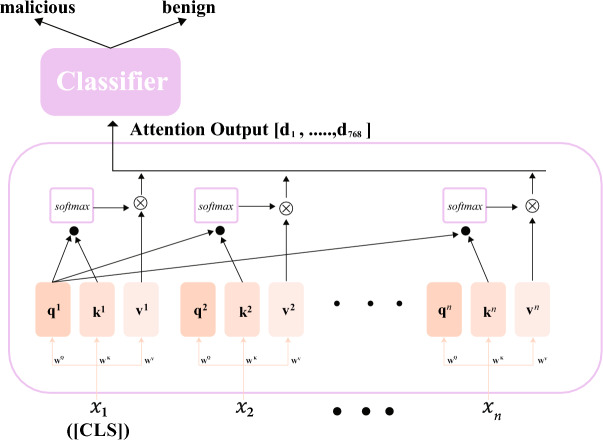
Self-attention mechanism and fine-tuning process.

The self-attention mechanism in BERT processes the URL string effectively by focusing on different sections simultaneously, capturing both local and global dependencies. It calculates attention scores for each token’s relevance to others, with the final representation encapsulated by the [CLS] token. Figure 5 visualizes the process of the self-attention mechanism and fine-tuning of the BERT model as it processes input tokens.

$X = [x_{1}, x_{2}, \ldots , x_{n}]$ is the collection of tokens in a URL that includes [CLS], as Fig. 5 illustrates. Using three metrics of weights-$W^{{\mathop {}\limits ^{Q}}}$, $W^{{\mathop {}\limits ^{K}}}$, and $W^{{\mathop {}\limits ^{V}}}$-each token ($x_{i}$), for $1 \le i \le n$, is projected onto vector space and multiplied to produce the corresponding triplets ($q_{i}$, $k_{i}$, and $v_{i}$), as shown in Eqs. 1, 2, and 3, respectively. A learning procedure is used to derive all three matrices, $W^{{\mathop {}\limits ^{Q}}}$, $W^{{\mathop {}\limits ^{K}}}$, and $W^{{\mathop {}\limits ^{V}}}$. The $q_{i}$ is the query, the $k_{i}$ is the key subject to retrieval, and the $v_{i}$ stands for the information included in the token. The ratios by which token $x_{1}$ should be impacted by all other tokens are then determined utilizing the Softmax function.

1 $$\begin{aligned} & q_{i} = x_{i} \cdot W^{{\mathop {}\limits ^{Q}}} \end{aligned}$$

2 $$\begin{aligned} & k_{i} = x_{i} \cdot W^{{\mathop {}\limits ^{K}}} \end{aligned}$$

3 $$\begin{aligned} & v_{i} = x_{i} \cdot W^{{\mathop {}\limits ^{V}}} \end{aligned}$$

Observe that Eq. 4, where $Q = [q_{1}, q_{2}, \ldots , q_{n}]$, $K = [k_{1}, k_{2}, \ldots , k_{n}]$, $V = [v_{1}, v_{2}, \ldots , v_{n}]$, and $\sqrt{d_{K}}$, specifies the dimensions of key $k$, the outputs for every input token $x_{i}$ are computed in parallel for $1 \le i \le n$. Each row of the self_attention output matrix represents token embeddings, capturing their meaning, position, and interactions based on non-zero softmax scores. With a maximum input length of 512 tokens, the 768-dimensional output vector reflects these attention scores.

4 $$\begin{aligned} \text {self}\_\text {attention}(Q, K, V) = \text {softmax} \left( \frac{QK^T}{\sqrt{d_k}} \right) V \end{aligned}$$

To sum up, the self-attention mechanism separates the URL into tokens and calculates contextual connections using attention scores, as represented by Eqs. 1–3. The overall semantic meaning is then extracted with Eq. 4 in order to ascertain whether the URL is malicious.

Subsequently, the attention layer’s outputs are then fed into the FNN within the encoder to enhance token representations through non-linear transformations. Each token’s features are processed independently with ReLU activation, as shown in Eq. 5, where ($x_{i}$) corresponds to the value representing token $i$ in the input sequence.

5 $$\begin{aligned} \text {ReLU}(x_{i}) = \text {max}(0,x_{i}) \end{aligned}$$

This enables the model to capture complex patterns needed for identifying malicious URLs. The FNN projects token representations to a higher-dimensional space and back, aiding in distinguishing URL categories. Its objective is to process the output from one attention layer so that it is suited as input for the next attention layer.

Ultimately, a classifier was incorporated into the encoder layer output for fine-tuning, as illustrated in Fig. 5. The chosen classifier for this study was Logistic Regression, a statistical method known for its effectiveness in binary classification tasks. This design choice was prioritized over a dense softmax layer to maintain computational efficiency during the frequent retraining cycles required by the adaptive pipeline. It further enables linear, interpretable decision boundaries for high-dimensional BERT embeddings. Logistic regression applies the sigmoid function to a combination of input features $x$ to model the probability of a binary outcome, mapping the weighted sum of input features to a value between 0 and 1, as described in Eq. 6.

6 $$\begin{aligned} \text {Sigmoid}(x) = \frac{1}{1+e^{-x}} \end{aligned}$$

In this configuration, logistic regression utilizes BERT-extracted features to classify URLs as either benign or malicious. The model fine-tunes BERT for binary classification by incorporating a classifier layer on top of the pooled output from the pre-trained BERT model. Upon completion of training, the model is capable of distinguishing between benign and malicious URLs.

### Adaptive retraining phase

In the adaptive retraining phase, the model undergoes systematic updates to adapt to new data and evolving patterns, ensuring relevance and effectiveness as data distributions change. This phase is crucial for ensuring that the model remains relevant and effective in real-world applications, where data distributions and features may change over time by cybercriminals.

#### Data collection

The initial stage in this phase is to collect new data samples, particularly from URLhaus, which provides hourly updates on malicious URLs, accessed through its API. This ensures the BERT model is periodically retrained with up-to-date data, improving its ability to distinguish between malicious and benign URLs. Listing 1 demonstrates the API requests utilized to collect the newly recorded malicious URLs.

**Listing 1:**
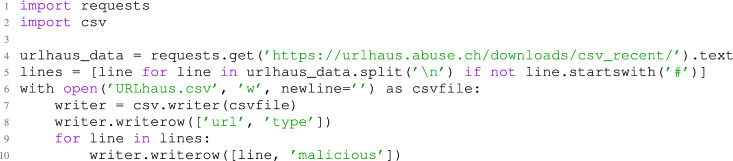
Fetching recent data from URLhaus API

The code snippet exhibits the utilization of the URLhaus API to fetch and prepare data by parsing and filtering out comments, and labeling URLs as ’malicious.’ The URLs and labels are stored in a CSV file and then merged with the existing dataset. Pre-processing, including data cleaning, ensures balance and originality. This approach continually enriches the dataset, enhancing the BERT model’s ability to differentiate URL categories.

#### Retraining BERT model

The model is retrained on the unified dataset, updating parameters to incorporate both old and new data, preserving previous knowledge while adapting to new patterns and features. During retraining, L1 regularization is applied to prevent overfitting by penalizing large model coefficients. This encourages feature selection, ensuring the model generalizes well to new data and retains previously learned patterns.

Continuous performance monitoring identifies any degradation, allowing adjustments to hyperparameters like learning rate and regularization. The retraining process is iterative, repeating these steps whenever new data becomes available or at predetermined intervals to keep the model adaptive and retain past knowledge.

### Testing phase

This phase focuses on evaluating the model’s performance in real-world scenarios, assessing its accuracy in classifying QR codes and its effectiveness in defending users against potential threats.

#### QR code scanning and URL extraction

The process begins with scanning the QR code. Using suitable software or libraries, the QR code image is captured either through a camera feed or by loading a pre-existing image file. Once the QR code is successfully scanned, the next step involves extracting the URL encoded within it. This extracted URL serves as the input for further analysis.

#### URL pre-processing

Before the URL can be analyzed, pre-processing steps are applied, including tokenizing the URL into manageable components. With the pre-processed URL in hand, the next step is to extract features using the pre-trained BERT model.

#### Pre-trained BERT model

The pre-trained BERT model is utilized to extract contextual embeddings or representations of the URL from its tokenized sequence. These features encapsulate the semantic information encoded within the URL, providing a rich representation for deeper examination. Subsequently, the extracted features are passed through a classification model trained on top of BERT.

#### Classification outcomes

The classification model is designed to distinguish between benign and malicious URLs based on their extracted features. Upon processing the features, the model produces predictions regarding the classification of the URL. The predicted classification, whether benign or malicious, is then presented to the user. For benign URLs, users may proceed with accessing the URL in a web browser. However, for URLs classified as malicious, a warning message is displayed, and access to the URL is restricted to prevent potential harm.

### Experimental setup

In this experiment, the BERT model was trained using a 20% subset of the data, which was not seen during training on the remaining 80% of the data. All hyperparameters used for this experiment employing the BERT model are detailed in Table 3.

**Table 3:** Hyperparameter settings for the BERT model.

Parameter	Value
BERT model	BERT base uncased
Maximum position embeddings	512
Batch size	512
Number of hidden layers	12
Hidden size	768
Number of attention heads	12
Regularization	L1
Solver	Saga
Regularization strength	C = 1.0

The experiment was conducted employing the Python programming language. All necessary libraries and packages were installed to ensure the successful execution of the code. The experimentation setup involved a computer equipped with an Intel Core i7 CPU boasting 32GB of memory, complemented by a GTX 1660 SUPER with 6GB of dedicated memory.

### Outcomes evaluation

In assessing the efficacy of the proposed BERT model, a confusion matrix is utilized to evaluate its performance by calculating True Positives (TP), True Negatives (TN), False Positives (FP), and False Negatives (FN). Table 4 demonstrates the representation of each variable.

**Table 4:** Confusion matrix for URL classification.

		Predicted label	
		Benign	Malicious
Actual Label	Benign	TN correctly classified as benign	FP incorrectly classified as malicious
	Malicious	FN incorrectly classified as benign	TP correctly classified as malicious

The confusion matrix counts are used to calculate performance metrics like accuracy, precision, recall, and F1 score. Accuracy, denoted in Eq. 7, measures the overall correctness of the classification model. It quantifies the ratio of correctly classified URL samples to the total number of URL samples in the dataset.

7 $$\begin{aligned} Accuracy = \frac{TP+TN}{TP+FP+TN+FN} \end{aligned}$$

Precision measures the proportion of correctly classified malicious URLs among all URLs predicted as malicious, as shown in Eq. 8.

8 $$\begin{aligned} Precision = \frac{TP}{TP+FP} \end{aligned}$$

Equation 9 illustrates recall measuring the proportion of correctly classified malicious URLs among all actual malicious URLs, also known as True Positive Rate (TPR).

9 $$\begin{aligned} Recall = \frac{TP}{TP+FN} \end{aligned}$$

The F1 score, represented by Eq. 10, is the harmonic mean of precision and recall and provides a balanced assessment between them.

10 $$\begin{aligned} F1\ Score = 2\times \frac{Precision\times Recall}{Precision+Recall} \end{aligned}$$

Macro Average was utilized to compute the mean value of these metrics for each category, ensuring unbiased findings despite imbalances across data categories. Furthermore, to evaluate model performance across varying thresholds, the area under the Receiver Operating Characteristic (ROC) curve (AUC ROC) was employed to quantify the trade-off between TPR and FPR across different classification thresholds. The AUC ROC value spans from 0 to 1, where 1 denotes a perfect classifier, and 0.5 indicates random guessing. TPR, commonly referred to as recall, denotes the proportion of malicious URLs that are accurately classified as malicious. A high TPR indicates effective identification of most malicious URLs while minimizing misclassification. On the other hand, FPR denotes the proportion of benign URLs incorrectly classified as malicious, calculated by Eq. 11. A low FPR signifies minimal misclassification of benign URLs as malicious.

11 $$\begin{aligned} FPR = \frac{FP}{FP+TN} \end{aligned}$$

## Experimental results analysis

The Results section examines the outcomes from initial BERT training and adaptive retraining, analyzing the model’s performance and effectiveness across various metrics.

### Training phase: BERT model evaluation

The performance of the BERT model is assessed using the Kaggle dataset^38^, which comprises two distinct types of URLs: benign and malicious. In this experiment, a subset of the data is extracted, with the amount of entries recorded per category being roughly 10,075 for benign URLs and 9,925 for malicious URLs, totaling 20,000 URL instances.

**Fig. 6:**
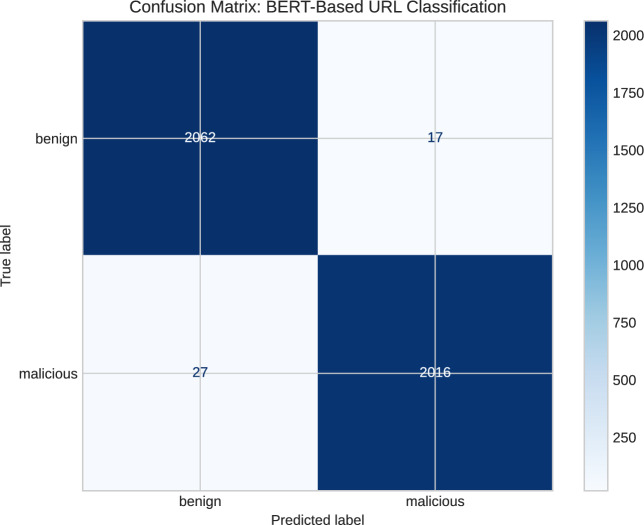
A confusion matrix of BERT model utilizing Kaggle dataset.

The outcomes are summarized in Table 5, demonstrating a training accuracy rate of 99.3% and a testing accuracy rate of 98.9%. To gain deeper insights into the model’s effectiveness, a confusion matrix was constructed using 20% of the dataset, comprising over 4,000 URL instances, as illustrated in Fig. 6.

**Table 5:** Performance analysis of BERT on Kaggle dataset.

Class	Precision	Recall	F1-score	Accuracy	TPR	FPR
Benign	0.987	0.992	0.989	0.989	0.987	0.008
Malicious	0.992	0.987	0.989			
Macro average	0.989	0.989	0.989			

Misclassifications in the confusion matrix can increase security risks, such as treating a malicious URL as benign. Therefore, the objective is to minimize these misclassifications to improve the model’s reliability and efficiency.

The AUC ROC metric, reported as 0.999, signifies the exceptional discriminative ability of the classifier in distinguishing between benign and malicious URLs. The model showcases consistently high TPRs alongside exceptionally low FPRs. This indicates the classifier’s competence in effectively identifying the majority of malicious URLs while maintaining minimal misclassifications of benign URLs.

### Adaptive retraining phase: outcome analysis

The adaptive retraining process incorporated regular updates, consisting of two distinct runs for this experiment, both sourcing data from the URLhaus API. As previously noted, URLhaus continuously records malicious URLs contributed by authenticated users on an hourly basis. Thus, this experiment emphasizes the importance of frequent data updates obtained through API crawling within specified time frames.

#### First round of retraining

In the initial round of retraining, 4714 URLs were gathered, comprising both newly recorded malicious URLs within a 48-h timeframe and benign URLs from other sources like Kaggle. These were then integrated with the original dataset, resulting in a total of 24,714 URLs, including 12,714 malicious URLs and 12,000 benign URLs, that were subsequently utilized to train the BERT model.

**Fig. 7:**
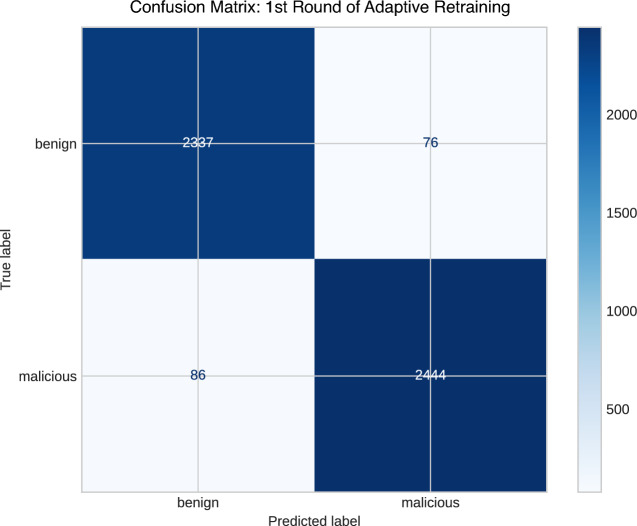
A confusion matrix of the first round in adaptive retraining phase.

Following the model’s retraining in this round, which resulted in an overall training accuracy of 97.7% and a testing accuracy of 96.7%, the findings of other metrics are elaborated in Table 6. Furthermore, the confusion matrix is depicted in Fig. 7.

**Table 6:** Performance analysis of the first round in adaptive retraining phase.

Class	Precision	Recall	F1-Score	Accuracy	TPR	FPR
Benign	0.964	0.968	0.965	0.967	0.967	0.031
Malicious	0.970	0.966	0.968			
Macro average	0.967	0.967	0.967			

The model achieved an impressive AUC ROC of 0.994, indicating its strong capability to distinguish benign URLs from malicious ones. This exceptional score underscores the model’s effective balance in detecting true positives while minimizing false positives.

#### Second round of retraining

In the second round of retraining, a total of 714 new URLs were gathered within a 24-h timeframe, sourced from URLhaus, along with additional benign URLs from various sources. Integrated with the initial training data, this resulted in a dataset totaling 25,428 URLs, encompassing 13,464 malicious URLs and 11,964 benign URLs.

**Fig. 8:**
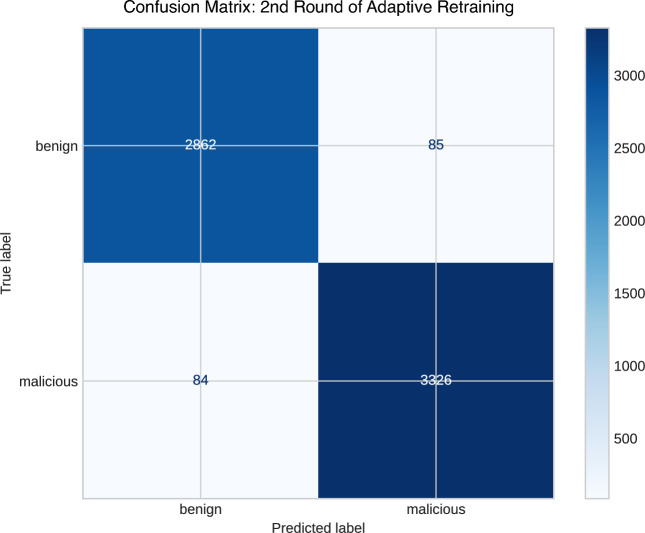
A confusion matrix of the second round in adaptive retraining phase.

Table 7 showcases the evaluation metrics, with a training accuracy of 97.4% and a testing accuracy of 97.3%. Additionally, Fig. 8 provides the confusion matrix summarizing the TPs and FPs outcomes across both categories.

**Table 7:** Performance analysis of the second round in adaptive retraining phase.

Class	Precision	Recall	F1-Score	Accuracy	TPR	FPR
Benign	0.971	0.980	0.975	0.973	0.975	0.028
Malicious	0.975	0.975	0.975			
Macro average	0.973	0.973	0.973			

In the second round of retraining, the model achieved a high AUC ROC of 0.994, reflecting its high proficiency in distinguishing benign from malicious URLs.

## Discussion

The previous results from the proposed BERT model reveal several noteworthy insights. Firstly, the model achieved a commendably high accuracy rate of 98.9%, underscoring its proficiency in distinguishing between benign and malicious URLs. This was further supported by the strong discrimination exhibited by the model, as evidenced by the high AUC ROC score of 0.999. A visual analysis of the model’s performance across multiple thresholds was offered by the ROC curve, demonstrating its ability to effectively balance true positives and false positives. Notably, these results remained consistent across multiple rounds of retraining, highlighting the model’s reliability and stability. Even with the addition of new data during retraining, the model’s performance remained robust, suggesting its adaptability to evolving threats. Consequently, these findings underscore the efficacy of the BERT model in URL classification tasks and its potential to bolster cybersecurity measures.

### Comparison with existing literature

The objective of the comparison with related works is to assess the effectiveness of the proposed BERT model within the context of existing studies on URL classification leveraging pre-trained language models and other baseline methods. Table 8 presents an overview of previous studies, including their approaches and results.

**Table 8:** Comparative analysis with related previous studies.

Research	Approach	Accuracy (%)
^11^	Transformer-based model	97.3
^3^	ML-XGBoost	96
^12^	BERT-based model	98.7
^27^	DL-FNN	95.9
^30^	Ensemble-based model	96.8
This study	Fine-tuned BERT model + classifier	**98.9**

The proposed BERT model outperforms other models, demonstrating superior performance in distinguishing between benign and malicious URLs. Its effectiveness stems from its capacity to capture intricate patterns and semantic nuances in URLs. Moreover, integrating Logistic Regression into the linear layer of BERT, coupled with L1 regularization, results in higher accuracy and more efficient performance. The model’s significance lies in setting new standards for URL classification, advancing the field of cybersecurity and large language modeling.

Regarding deployment feasibility, while transformer-based models incur higher computational costs than lightweight lexical parsers, the inference latency of the base-BERT architecture remains within acceptable thresholds for real-time scanning applications, particularly when deployed on cloud-edge architectures. Furthermore, BERT is utilized here as a foundational encoder to validate the efficacy of the adaptive retraining pipeline itself. The primary contribution lies in the system’s dynamic capability to address concept drift, a methodology that is model-agnostic and can be extended to other transformer backbones.

### Limitations and challenges

Despite the promising results demonstrated by the proposed framework, several limitations regarding computational overhead and resource scalability must be acknowledged. Primarily, the reliance on a transformer-based architecture necessitates enhanced computational resources to efficiently process large-scale datasets. Furthermore, as the system undergoes adaptive retraining, the continuous accumulation of data volume requires a robust server infrastructure to manage the expanding collection of URLs effectively. A critical challenge also lies in memory management, where sufficient allocation is required to retain historical patterns during updates, preventing knowledge loss without the computational burden of full-dataset retraining.

## Conclusion

In this study, a novel approach utilizing BERT for non-natural language processing tasks is presented, focusing on detecting embedded malicious URLs within QR codes. To ensure the adaptability of the proposed approach, periodic model refinement has been implemented by retraining the model on newly evolved malicious URLs to counter the latest tactics utilized by cybercriminals. Extensive tests conducted on two different public datasets, Kaggle and URLhaus, employed for initial training and adaptive retraining phases, have proven the efficacy of the proposed BERT model. Kaggle was extensively utilized during the initial training phase, resulting in an impressive accuracy rate of 98.9% and an excellent AUC ROC score of 0.999. Two rounds of retraining were conducted in the experiment, demonstrating the model’s competence in accurately adapting to newly evolved patterns. The results demonstrate a significant advancement in classification accuracy relative to previous methodologies. Furthermore, the proposed pre-trained model has been tested with a set of QR code inputs, confirming its reliability and efficacy in identifying malicious URLs embedded within QR codes. This prevents users from being redirected to potentially harmful content upon detection of an unsafe URL. Limitations comprise the need for powerful computational resources and memory allocation to manage larger datasets and retain learned patterns during the adaptive retraining phase. Future research advancements involve integrating the proposed BERT model with methods for verifying the HTML content of webpages to further enhance user safety upon scanning QR codes. Additionally, exploring alternative classifier algorithms such as Random Forest and Neural Networks, along with suitable datasets, is suggested for further improvements.

## Data Availability

The data supporting the findings of this study can be provided by the corresponding author upon request.
